# Correction: Dicopper(i) complexes of a binucleating, dianionic, naphthyridine bis(amide) ligand

**DOI:** 10.1039/d5dt90059j

**Published:** 2025-03-21

**Authors:** Laurent Sévery, T. Alexander Wheeler, Amelie Nicolay, Simon J. Teat, T. Don Tilley

**Affiliations:** a Department of Chemistry, University of California, Berkeley Berkeley CA 94720-1460 USA tdtilley@berkeley.edu; b Chemical Sciences Division, Lawrence Berkeley National Laboratory Berkeley CA 94720 USA; c Advanced Light Source, Lawrence Berkeley National Laboratory Berkeley CA 94720 USA

## Abstract

Correction for ‘Dicopper(i) complexes of a binucleating, dianionic, naphthyridine bis(amide) ligand’ by Laurent Sévery *et al.*, *Dalton Trans.*, 2025, https://doi.org/10.1039/d5dt00034c.

Fig. 5 in the original submission contained an erroneous legend. The figure is provided here with the corrected legend using the appropriate naming conventions of the compounds discussed in the original text.

**Fig. 1:**
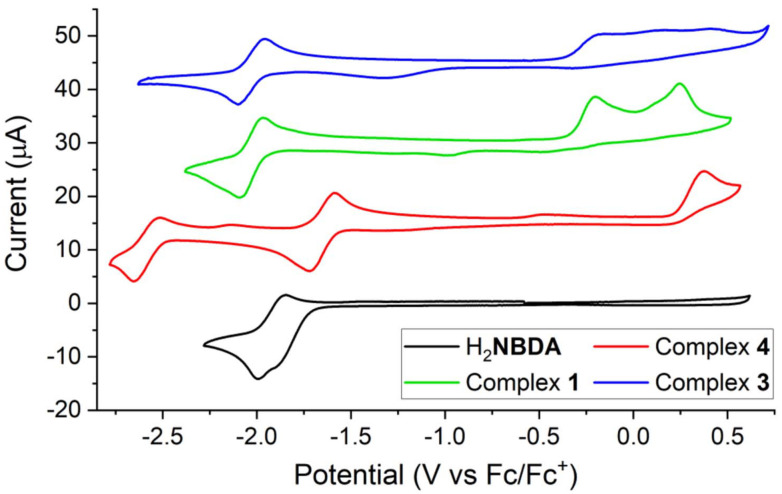
CVs of H_2_**NBDA** and the corresponding dicopper(i) **NBDA** complexes **1**, **3** and **4**. Conditions: 100 mV s^−1^ scan rate; GC working electrode, Pt counter electrode, Ag/AgBF_4_ reference electrode; 0.1 M Bu_4_NPF_6_ in THF.

The Royal Society of Chemistry apologises for these errors and any consequent inconvenience to authors and readers.

